# Postictal status and clinical variables in adults with epilepsy

**DOI:** 10.1055/s-0045-1811625

**Published:** 2025-09-08

**Authors:** Cecília Fusco Agostinho, Glória M. A. S. Tedrus

**Affiliations:** 1 Pontifícia Universidade Católica de Campinas, Campinas SP, Brazil.

**Keywords:** Epilepsy, Seizures, Confusion

## Abstract

**Background:**

Symptoms in the postictal period are often neglected by professionals and patients/family members.

**Objective:**

To relate the characteristics of the postictal period with the clinical variables of adult patients with epilepsy.

**Methods:**

Prospectively, the clinical characteristics of the postictal period were related to the clinical variables and the scores on the Hospital Anxiety and Depression Scale (HADS) and Mini-Mental State Examination (MMSE) of 70 patients with epilepsy.

**Results:**

The mean age was 47.6 years old, and 34 (48.6%) patients were male, the mean age at onset of epilepsy was 20.3 years old, the epileptic syndrome was structural in 48 cases, the mean score in the MMSE was 23.6, the mean score in the HADS-anxiety was 7.3, and in the HADS-depression it was 5.6. Postictal manifestation occurred in 59 (84.2%) cases, with prolonged duration in 22 (37.2%) cases. There was a difference in the duration and presence of postictal manifestation according to age at the onset of epilepsy. Higher HADS-anxiety scores were associated with the presence of confusion in the postictal period (t-test; 9.3 ± 6.8 versus 5.8 ± 4.8; *p =* 0.015). Lower MMSE scores were related to postictal anxiety and sleepiness/headache/confusion. Left-sided epileptiform activity (EA) was associated with postictal sleepiness/headache/confusion. There was a difference in postictal manifestation according to epileptic syndrome.

**Conclusion:**

The characteristics of the postictal period differed according to the age of onset, the type of seizure, and the epileptic syndrome. Different symptoms in the postictal period were associated with the scores on HADS anxiety, MMSE, and left-sided EA on the electroencephalogram.

## INTRODUCTION

The postictal period is a condition that appears between the end of the ictal manifestation and the return to the initial condition (presumed baseline) or the interictal state. 1 2 The postictal period is a transient brain condition subject to spontaneous resolution. It presents a series of neurological and psychiatric signs and symptoms with varying severity and variable duration, from minutes to several days. 1 3 The postictal period is the phase after the ictal manifestation; it is accompanied by slowing or depression of brain electrical activity on the electroencephalogram (EEG) that can last up to 24 hours. The postictal period results from complex neurophysiological mechanisms such as hypometabolism, changes in blood flow and glucose metabolism, and reduced cortical excitability and neurotransmitter release. 4 5 6 7

The postictal period can significantly impact the quality of life, but it is often neglected by patients/family members and attending physicians. 8 9 However, the description of the relationship between the clinical manifestations of the postictal period and the characteristics of epilepsy is still scarce and has often not received due attention in the current literature.

The objective of the present study was to evaluate the clinical manifestations of the postictal period to investigate the related clinical variables in adult patients with epilepsy.

## METHODS

A prospective observational cohort from July 2023 to March 2024 included 70 adult patients diagnosed with structural focal epilepsy or of unknown etiology, according to the International League Against Epilepsy (ILAE) criteria, 10 diagnosed at least 2 years ago, in regular use of antiseizure medication (ASM) and treated at Hospital PUC-Campinas, Campinas, state of São Paulo, Brazil.

Patients with severe cognitive impairment, disabling and progressive chronic diseases, and genetic generalized epilepsy were excluded.

All patients gave written informed consent, and the Human Ethics Committee approved the present project (CAAE: 59698922.5.0000.5481. no.: 5.507.182).

### Procedures

All included patients were evaluated individually in the clinical neurology outpatient clinic rooms. Demographic and clinical data and imaging test results were collected (magnetic resonance imaging [MRI] and cranial tomography). On resting EEG, the presence and laterality of epileptiform activity were observed. Age at onset of epilepsy, current seizure type and frequency, and ASM use were observed. Seizures were categorized by the manifestation of their onset (focal or generalized onset). Ictal and postictal period characteristics were obtained from the patients and their families. The end of critical clinical activity was considered to characterize the beginning of the postictal phase. In the postictal period, the presence of mental, emotional, autonomic, and motor symptoms and signs was evaluated, as well as the time until full recovery ("return to normal").

The Mini-Mental State Examination (MMSE) was used for cognitive screening, 11 12 and the Hospital Anxiety and Depression Scale (HADS) 13 14 was used to assess the presence of depressive and anxious symptoms.

### Data analysis

The characteristics of the postictal period (duration and type of symptoms), the clinical variables of epilepsy, the laterality of epileptiform activity on the EEG, and the MMSE and HADS scores were related. The present study considered the prolonged postictal period when the symptoms lasted > 30 minutes. The total MMSE score was evaluated according to formal schooling in the years of study according to the Brazilian validation. It was classified as low (cognitive impairment) and normal (no cognitive impairment) scores. For the evaluation of the HADS, the cutoff score was used according to the criteria validated in Brazil (HADS-anxiety ≥ 8 HADS-depression > 7). 14

A descriptive analysis of the variables was performed as the mean, minimum, maximum, and percentage values. The chi-squared test and the t-test were used for analysis. The factors associated with the postictal phase were evaluated using multiple logistic regression with a selection of stepwise variables. Statistical analysis was conducted using IBM SPSS statistics version 27.0 (SPSS, IBM Corp.), with a significance level of *p* < 0.05 *.*

## RESULTS

Demographic and clinical data and postictal period characteristics are shown in Table 1 .

**Table 1:** Demographic and clinical data and characteristics of the postictal period of 70 patients

		Average, (minimum–maximum) or %
Average age (years old)		47.6 (20–90)
Formal education (years)		9.0 (2–15)
Female gender ( *n* , %)		36 (51.4%)
Mean age at onset of epilepsy (years old)		20.3 (1–77)
Average duration of epilepsy (years)		27.4 (1–78)
Type of seizure: Focal/exclusively generalized (n, %)		52 (74.3%)/18 (25.7%)
Seizure frequency: ≥ 1x/month/1–11x/year/ no seizures in the year		21/22/27
EA laterality: right/left ( *n* , %)		7 (20%)/28 (80%)
No. of ASM in use: 1/≥2 ( *n* , %)		48 (68.6%)/22 (31.4%)
Epileptic syndrome: structural/unknown ( *n* , %)		48 (68.6%)/22 (31.4%)
MMSE total score		23.6 (10–29)
HADS-anxiety (total score)		7.3 (0–19)
HADS-depression (total score)		5.6 (0–20)
Characteristics of the postictal period	Present ( *n* , %)	59 (84.2%)
	Duration > 30 minutes	22 (37.2%)
Clinical manifestation	Drowsiness/altered states of consciousness ( *n* , %)	22 (31.4%)
	Headache ( *n* , %)	19 (27.1%)
	Mental confusion ( *n* , %)	30 (42.9%)
	Motor (including aphasia) ( *n* , %)	25 (35.7%)
	Changes in mood and affect ( *n* , %)	25 (35.7%)
	Other ( *n* , %)	38 (54.3%)

Abbreviations: ASM, anti-seizure medication; EA, epileptiform activity; HADS, Hospital Anxiety and Depression Scale; MMSE, Mini-Mental State Examination.

Postictal manifestation occurred in 59 (84.2%) cases and was present as a single symptom or as a cluster of symptoms. The patients who presented clinical manifestations in the postictal period had lower MMSE scores, had left-sided epileptiform activity on the EEG, and had a lower age at the occurrence of the first seizure. No difference was observed in the occurrence of the postictal period according to demographic aspects, the location of the epileptiform activity, and other epilepsy variables ( Table 2 ).

**Table 2:** Occurrence of clinical manifestation in the postictal period according to clinical aspects of epilepsy

		Postictal period		*p* - *value*
		*No (n = 11)*	*Yes (n = 59)*	
Age (years old)		54.6	46.3	0.071 ^a^
Age at onset of epilepsy (years old)		21.5	20.8	0.07 ^a^
Duration of epilepsy (years)		33.0	25.5	0.281 ^a^
Laterality of the EA on the EEG	Right ( *n* = 7)	3	4	0.01 ^ b *^
	Left ( *n* = 28)	1	27	
MMSE		25.3 ± 2.7	23.3 ± 3.4	0.047 ^a^ *
MMSE/schooling	Low score ( *n* = 40)	3	37	0.045 ^b^ *
	Normal score ( *n* = 30)	8	22	

Abbreviations: EA, epileptiform activity ***;*** EEG, electroencephalogram; MMSE, Mini-Mental State Examination.

Notes: ^a^ T-test; ^b^ Fisher's exact test; * *p* < 0.05 *.*

The clinical characteristics of the postictal period differed according to some epilepsy variables. There was an association between the type of seizure and epileptic syndrome, the laterality of the EA, the presence of anxiety symptoms (HADS), and cognitive deficits (MMSE total score), with different clinical manifestations in the postictal period ( Table 3 ). Language dysfunction (speech and language disturbances, dysphasia) in the postictal period occurred in 12 cases, but there was no association with epilepsy variables.

**Table 3:** Relationship between manifestations in the postictal period and epilepsy variables

Postictal period	Epilepsy variables		*p-value*
Length of the postictal period	Age at onset of epilepsy		
*< 30 minutes*	23.7 ± 19.6		0.014 ^a^ *
*≥ 30 minutes*	14.0 ± 9.5		
Headache	Age at onset of epilepsy		
*Yes*	23.7 ± 17.7		0.016 ^a^ *
*No*	13.7 ± 13.3		
Confusion	HADS-anxiety		
*Yes*	9.3 ± 6.8		0.015 ^a^ *
*No*	5.8 ± 4.8		
Changes in mood and affect	MMSE - Total score		
*Yes*	24.5 ± 2.5		0.014 ^a^ *
*No*	22.4 ± 3.7		
Drowsiness/headache/confusion	MMSE - Total score		
*Yes*	23.0 ± 3.6		0.039 ^a^ *
*No*	24.6 ± 2.7		
Sleepiness/altered states of consciousness	Szs: disperceptive/generalized	Perceptive	
*Yes*	21	1	0.041 ^b^ *
*No*	36	12	
Headache	Seizures -generalized	Focal	
*Yes*	12	36	0.017 ^b^ *
*No*	1	21	
Confusion	EEG - Right EA	EA Left	
*Yes*	0	16	0.007 ^b^ *
*No*	7	12	
Motor manifestation	Structural syndrome	Unknown	
*Yes*	20	5	0.041 ^b^ *
*No*	25	20	
Changes in mood and affect	MMSE - Low score	Normal score	
Yes	13	12	0.045 ^b^ *
*No*	9	25	
Drowsiness/headache/confusion	ELT-EH	Other epilepsies	
Yes	13	5	0.038 ^b^ *
*No*	11	16	
Drowsiness/headache/confusion	EEG - Right EA	EEG - Left EA	
*Yes*	2	20	0.036 ^b^ *
*No*	5	8	

Abbreviations: EA, epileptiform activity; EEG, electroencephalogram; HADS, Hospital Anxiety and Depression Scale; MMSE, Mini-Mental State Examination; Szs, seizures; TLE-HS, temporal lobe epilepsy with hippocampal sclerosis.

Notes: ^a^ T-test; ^b^ Chi-squared test; * *p <* 0.05 *.*

All variables that presented a *p* -value ≤ 0.2 in the individual analysis were placed in a multiple logistic regression model, with stepwise variable selection criteria. In the final model, no variable maintained statistical significance for predicting the chance of the postictal period.

## DISCUSSION

In the present study, there was a high occurrence of some symptoms with longer duration in the postictal period. In many cases, the duration of the manifestations was prolonged and presented as a cluster of symptoms. The importance of characterizing and better understanding this seizure phase may contribute to the understanding of the pathophysiological disorders involved with cerebral electrical shutdown, which is associated with postictal morbidity and mortality. 15 In addition, the study of the mechanisms involved in the postictal period contributes to the localization of the epileptogenic focus in the presurgical evaluation of epilepsy, in the differential diagnosis between epileptic and nonepileptic events, and in the assessment of the use of some ASM, vagus nerve stimulation, or the use of symptomatic medications in the management of these patients to improve self-esteem and quality of life.

### Epilepsy variables: symptoms/signs in the postictal period

It was observed that aspects of seizures/epilepsy were associated with different characteristics of the postictal period, similar to studies that describe the high frequency of cognitive, sensory, and autonomic disorders, headache, and changes in mood and affect and psychosis in the postictal period. 14 16 17 18 19

The occurrence of a cluster of symptoms in the postictal period was higher in patients who presented with cognitive impairment and left laterality of interictal epileptiform. The relationship between the postictal symptoms and hemispheric laterality epileptiform activity may be related to alterations in functional connectivity networks. Another study found no association between postictal manifestation and EEG and neuroimaging data when evaluating 100 patients. 18

The longer duration of the postictal period was associated with younger age at the onset of epilepsy but not with the patient's age, indicating the complexity of brain functional alterations and neuronal plasticity reserve caused by chronic and prolonged epilepsy. Similarly, studies have described prolonged duration in temporal lobe epilepsy with hippocampal sclerosis (TLE-HS) 7 20 and in TLE-HS with seizures of onset before 17 years of age. 21 There was no relationship between the length of the postictal period and type of seizure, similar to the findings of a recent systematic review. 17 In a study with 209 adults with generalized seizures, the association between the duration of this period, age ≥ 65 years old, the duration of the ictal period, and functional capacity was described. 22

There was an association between the presence of headache and longer duration of the postictal period, with epilepsy of later onset and with generalized seizures. It is known that headache is the most frequent symptom, occurring in ∼ 52% of cases, particularly in bilateral tonic-clonic seizures and in drug-resistant epilepsy. In TLE-HS, the location of the headache is ipsilateral to the onset of seizure. 22 23

Changes in mood and affect had a high occurrence, particularly in patients who had previous cognitive impairment. Negative psychic symptoms and postictal depressive episodes are relatively frequent, may be the only manifestation or be associated with other clinical manifestations, with a higher occurrence in patients with drug-resistant epilepsy, and may be characterized as an exacerbation of mood and anxiety disorders of the interictal period with a significant negative impact on quality of life. 8 9 18

In the present series, the presence of symptoms of mental confusion was frequent, similar to other studies. It is known that there are difficulties in distinguishing postictal confusion from other symptoms/signs, such as lethargy, specific cognitive disorders, and attention. Postictal unresponsiveness is described in a high number of cases. 17 The occurrence of postictal mental confusion was more frequent in patients with high HADS-anxiety scores, which confirms that postictal and interictal psychiatric symptomatology are closely related, implying common pathogenic mechanisms.

We did not observe a relationship between epilepsy variables and the presence of postictal language disorder. It is known that aphasia in the immediate postictal occurs in ∼ 38% of cases and may have localization value (localization and laterality), particularly in TLE-HS. In patients with postictal dysphasia, discharge originates in the dominant hemisphere in 92% of cases. 24

The cluster of symptoms drowsiness/headache/confusion was more frequent in TLE-HS, which may indicate that global or focal neurological impairment in epilepsy may predispose to specific postictal symptoms, as well as influence ictal phenomena and brain regions involved in epileptic discharge. 7

The presence of transient postictal motor manifestation (Todd's paresis) was more frequent in structural epilepsy. Todd's paresis occurs in 6 to 13% of seizures, has variable duration, is unilateral and contralateral to the focus, and occurs more frequently in cases with motor seizures, with structural lesions, and in older people, and is associated with perfusion deficit and metabolic demands due to seizures. 25

In conclusion, postictal symptoms were frequent, had different types of manifestation and duration, and were associated with several clinical variables, EEG data, and cognitive/psychiatric aspects.

### Limitations

There are several limitations in the present study. The study was carried out in a single center; there was no video recording of the seizures, and the data of the postictal period were obtained only with the clinical observation of the patients/relatives. Thus, they are prone to subjectivity and lack of precision, which may not have good accuracy. Another limitation observed in the present study was the use of a screening scale for the evaluation of psychiatric comorbidities (anxiety/depression) and cognitive deficits. Well-designed prospective studies are needed.
